# Development of a single, practical measure of surgical site infection (SSI) for patient self-report and observer completion: a novel method of questionnaire design

**DOI:** 10.1186/1745-6215-16-S2-O6

**Published:** 2015-11-16

**Authors:** Rhiannon Macefield, Alex Nicholson, Tom Milne, Thomas Pinkney, Melanie Calvert, Kerry Avery, Barnaby Reeves, Jane Blazeby

**Affiliations:** 1 University of Bristol, Bristol, UK; 2 University Hospitals Birmingham NHS Foundation Trust, Birmingham, UK; 3 University of Birmingham, Birmingham, UK; 4 University Hospitals Bristol NHS Foundation Trust, Bristol, UK

## 

Randomised controlled trials in surgery often aim to measure SSIs but accurate assessment is difficult, particularly after hospital discharge when most SSIs present. Commonly used SSI criteria and grading scales exist but require experienced observers, do not identify SSIs consistently and have not considered patients' perspectives. We aimed to develop a novel single measure for SSI, suitable for patient self-report and observer completion, for accurate and efficient outcome assessment for use in RCTs.

Content analyses of existing tools (applying CDC and ASEPSIS definitions) and semi-structured interviews with patients and professionals (n=19) identified SSI domains. Domains were used to inform item development using standard methods for designing patient questionnaires. Medical terms describing the underlying domains were included at the end of items in parentheses. Iterative cognitive interviews with patients (n=28) and professionals (ongoing) pre-tested face validity and acceptability of a single measure for SSI assessment.

Content analyses of existing tools identified 18 domains, supplemented by one additional domain identified from interviews. These were operationalised into a provisional questionnaire with 24 items. Cognitive interviews and discussions within the research team led to six iterations of the questionnaire including 16 changes to items, six changes to instructions and two changes to response categories. It was acceptable to patients to include medical terminology alongside lay terms.

A single SSI measure has been developed for patients and observers to provide outcome data for use in RCTs. Ongoing work will continue to examine the validity and reliability of this approach and its applicability to other settings.

